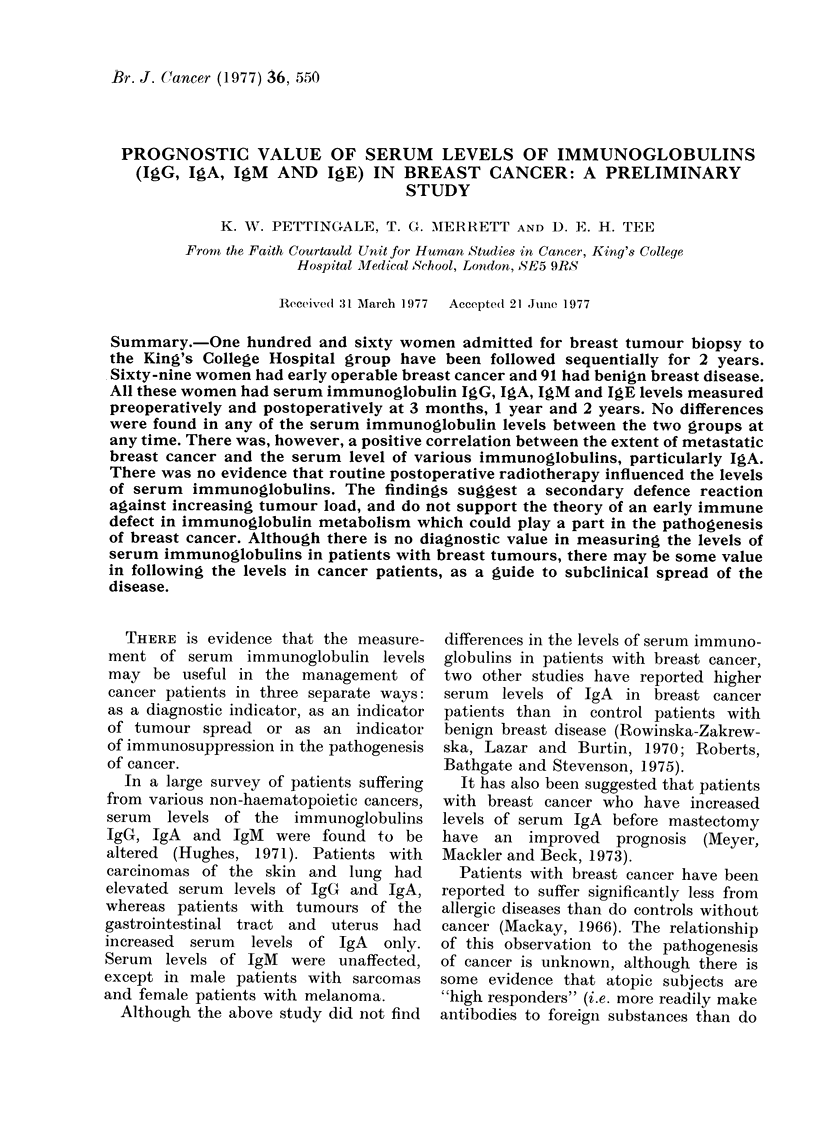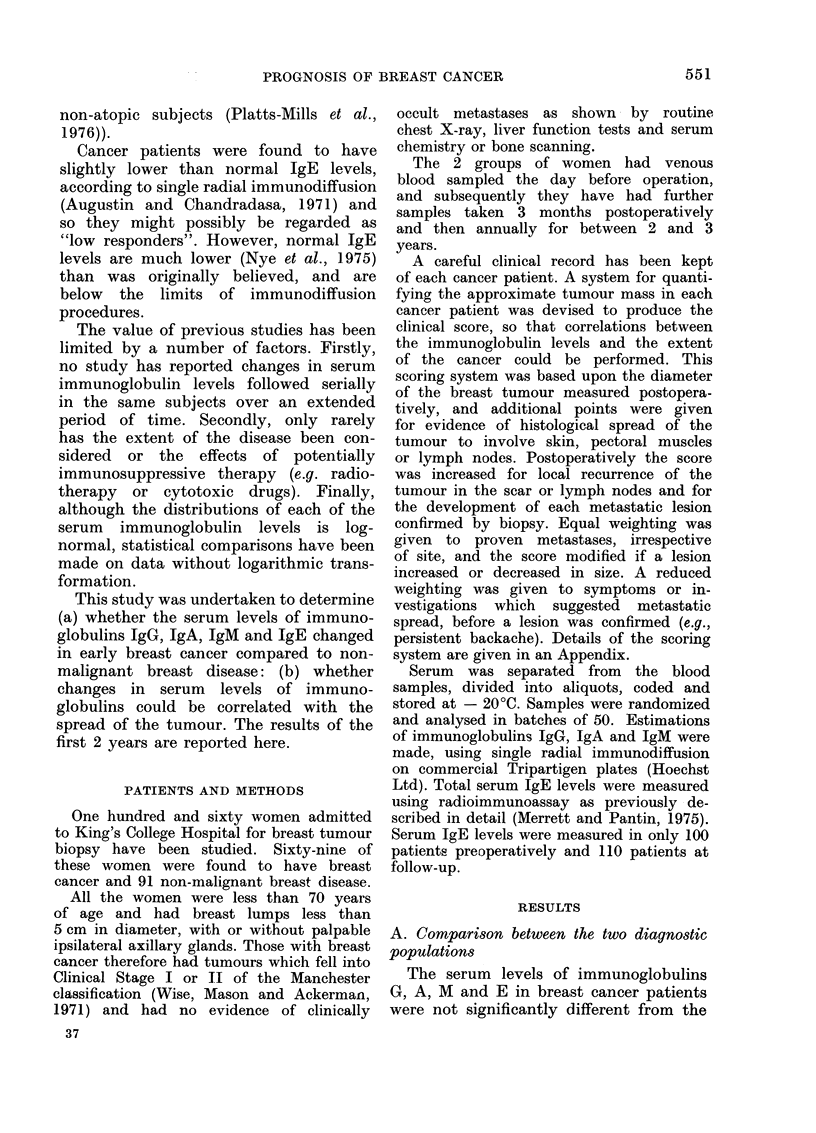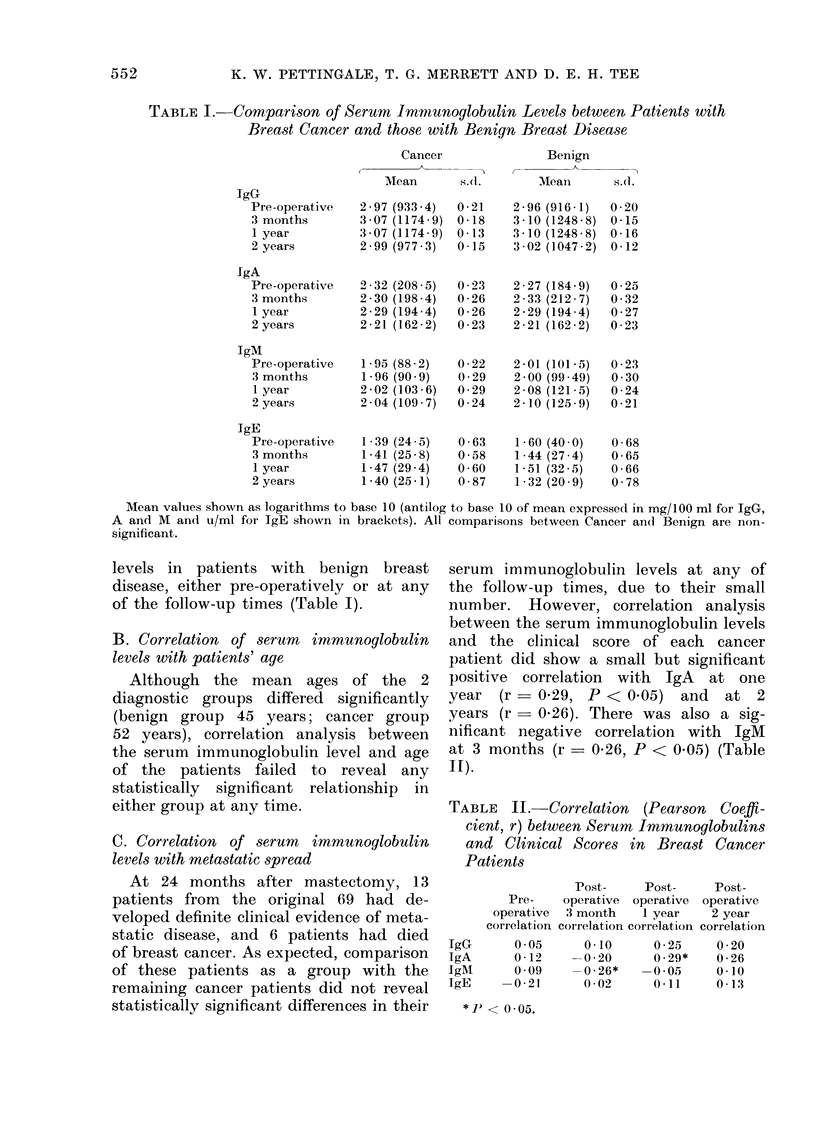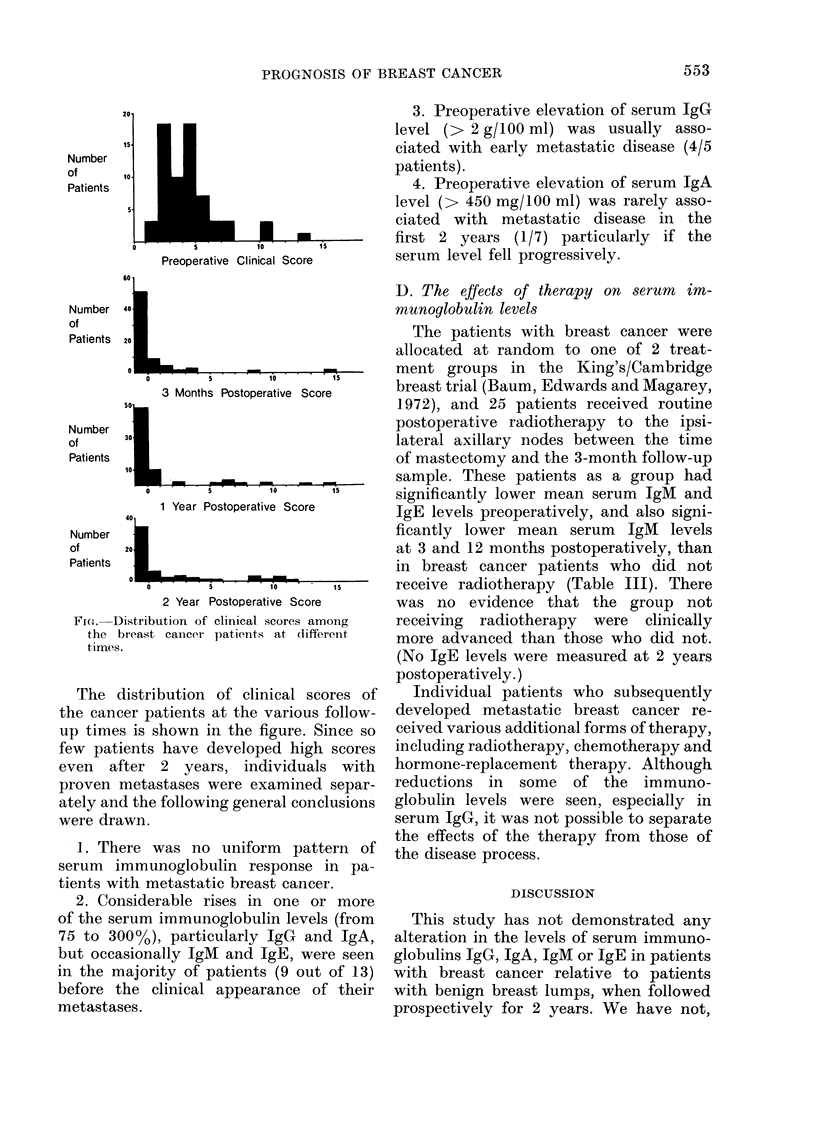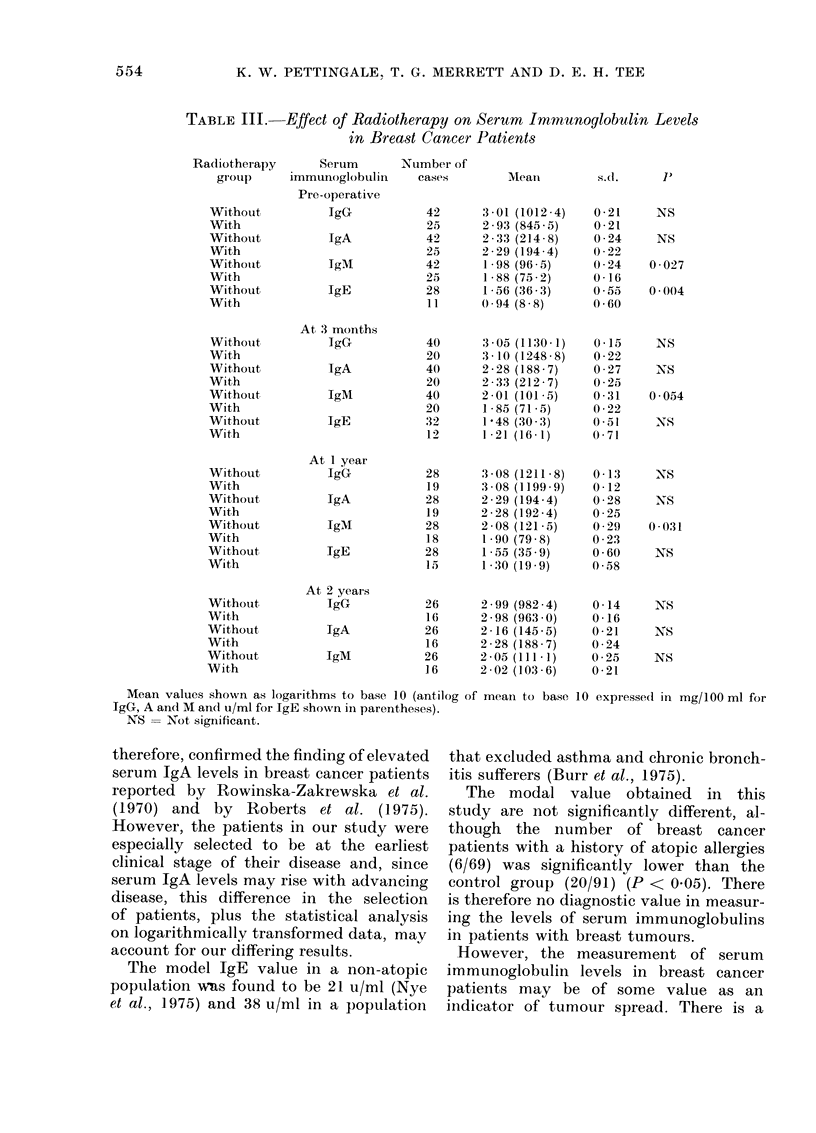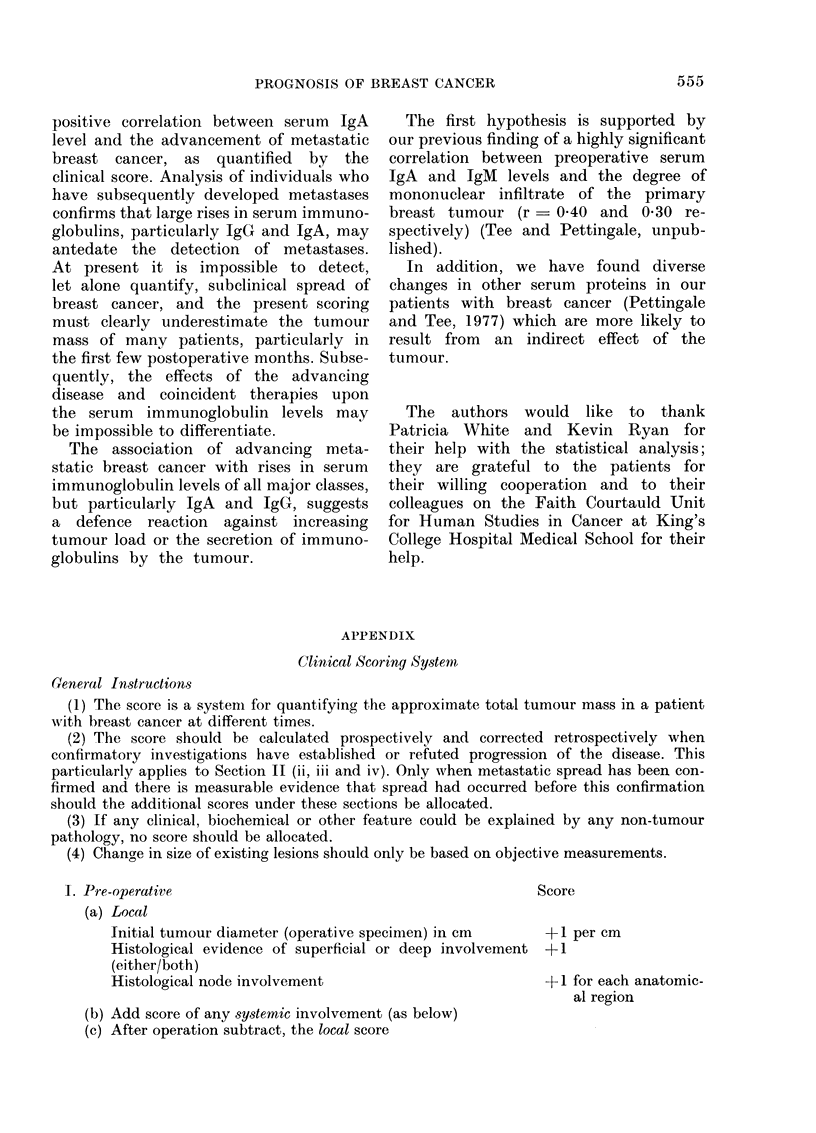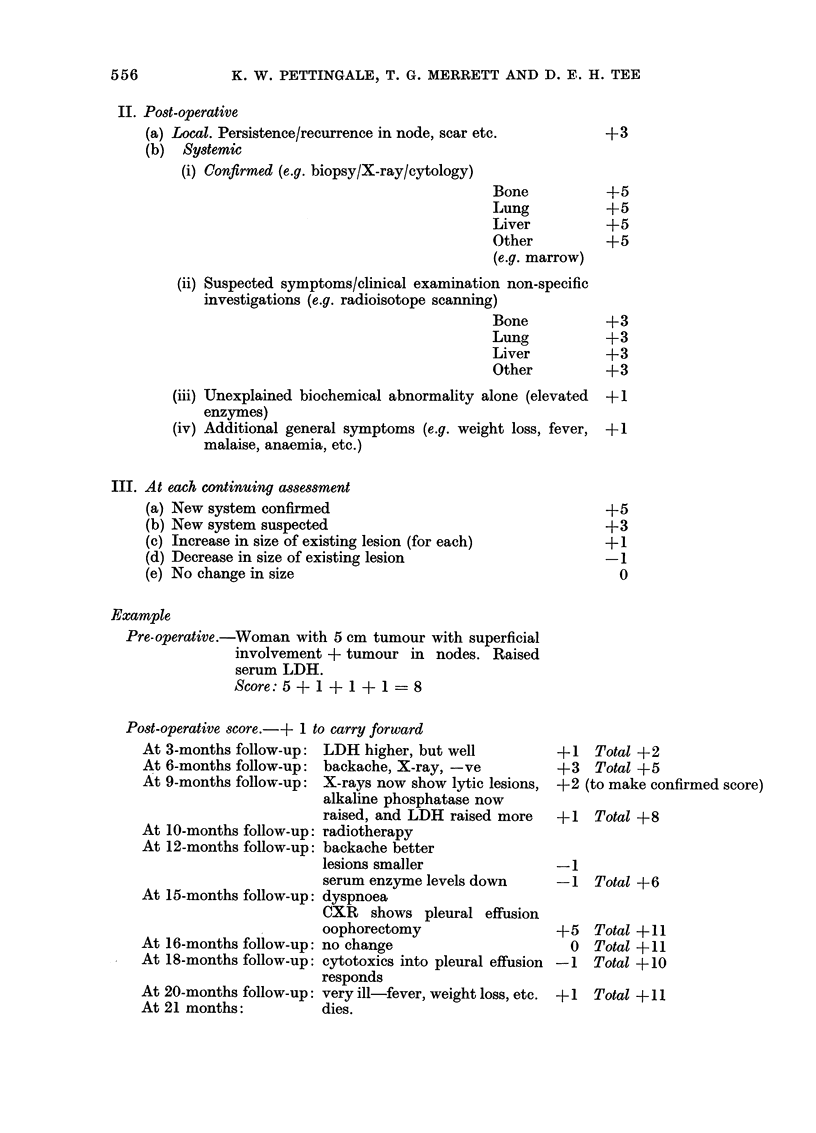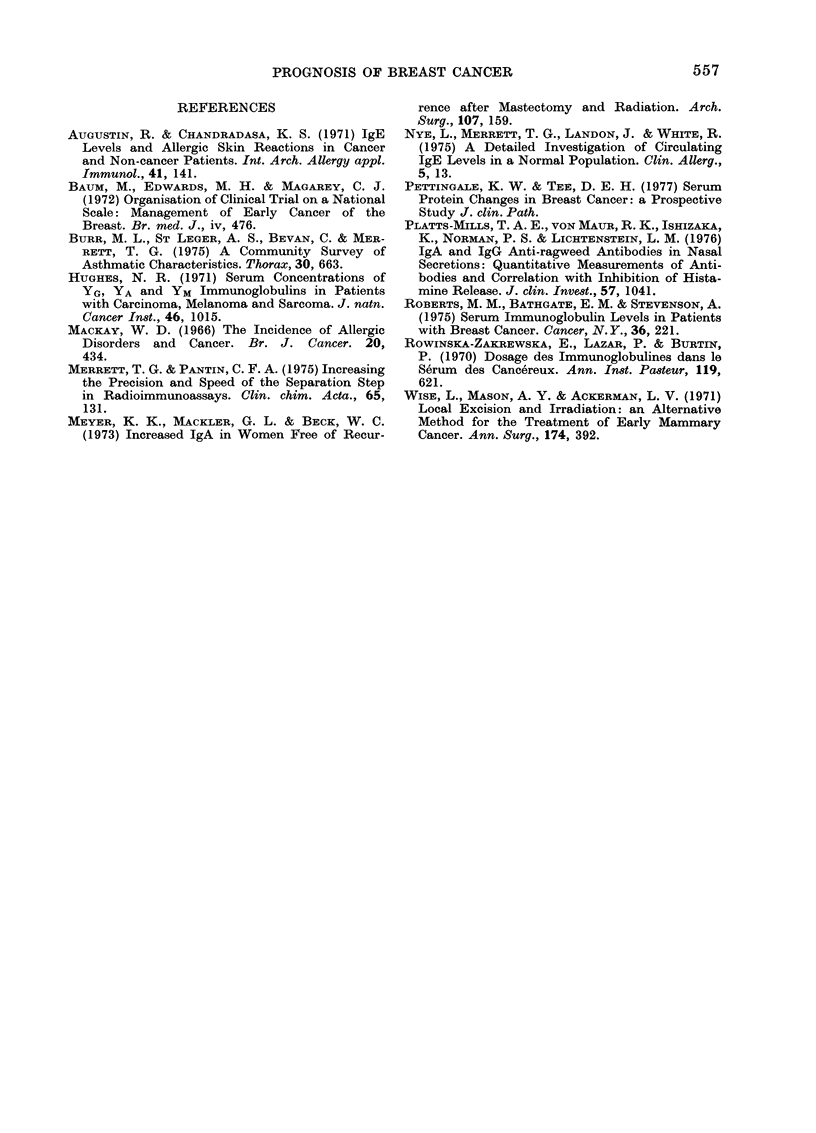# Prognostic value of serum levels of immunoglobulins (IgG, IgA, IgM and IgE) in breast cancer: a preliminary study.

**DOI:** 10.1038/bjc.1977.230

**Published:** 1977-11

**Authors:** K. W. Pettingale, T. G. Merrett, D. E. Tee

## Abstract

One hundred and sixty women admitted for breast tumour biopsy to the King's College Hospital group have been followed sequentially for 2 years. Sixty-nine women had early operable breast cancer and 91 had benign breast disease. All these women had serum immunoglobulin IgG, IgA, IgM and IgE levels measured preoperatively and postoperatively at 3 months, 1 year and 2 years. No differences were found in any of the serum immunoglobulin levels between the two groups at any time. There was, however, a positive correlation between the extent of metastatic breast cancer and the serum level of various immunoglobulins, particularly IgA. There was no evidence that routine postoperative radiotherapy influenced the levels of serum immunoglobulins. The findings suggest a secondary defence reaction against increasing tumour load, and do not support the theory of an early immune defect in immunoglobulin metabolism which could play a part in the pathogenesis of breast cancer. Although there is no diagnostic value in measuring the levels of serum immunoglobulins in patients with breast tumours, there may be some value in following the levels in cancer patients, as a guide to subclinical spread of the disease.


Br. J. Cancer (1977) 36, 550

### PROGNOSTIC VALUE OF SERUM LEVELS OF IMMUNOGLOBULINS (IgG, IgA, IgM AND IgE) IN BREAST CANCER: A PRELIMINARY STUDY

K. W. PETTINGALE, T. G. MERRETT AND D. E. H. TEE

*From the Faith Courtauld Unit for Human Studies in Cancer, King's College Hospital Medical School, London, SE5 9RS*

Received 31 March 1977 Accepted 21 June 1977

**Summary.—One hundred and sixty women admitted for breast tumour biopsy to the King's College Hospital group have been followed sequentially for 2 years. Sixty-nine women had early operable breast cancer and 91 had benign breast disease. All these women had serum immunoglobulin IgG, IgA, IgM and IgE levels measured preoperatively and postoperatively at 3 months, 1 year and 2 years. No differences were found in any of the serum immunoglobulin levels between the two groups at any time. There was, however, a positive correlation between the extent of metastatic breast cancer and the serum level of various immunoglobulins, particularly IgA. There was no evidence that routine postoperative radiotherapy influenced the levels of serum immunoglobulins. The findings suggest a secondary defence reaction against increasing tumour load, and do not support the theory of an early immune defect in immunoglobulin metabolism which could play a part in the pathogenesis of breast cancer. Although there is no diagnostic value in measuring the levels of serum immunoglobulins in patients with breast tumours, there may be some value in following the levels in cancer patients, as a guide to subclinical spread of the disease.**

THERE is evidence that the measurement of serum immunoglobulin levels may be useful in the management of cancer patients in three separate ways: as a diagnostic indicator, as an indicator of tumour spread or as an indicator of immunosuppression in the pathogenesis of cancer.

In a large survey of patients suffering from various non-haematopoietic cancers, serum levels of the immunoglobulins IgG, IgA and IgM were found to be altered (Hughes, 1971). Patients with carcinomas of the skin and lung had elevated serum levels of IgG and IgA, whereas patients with tumours of the gastrointestinal tract and uterus had increased serum levels of IgA only. Serum levels of IgM were unaffected, except in male patients with sarcomas and female patients with melanoma.

Although the above study did not find differences in the levels of serum immunoglobulins in patients with breast cancer, two other studies have reported higher serum levels of IgA in breast cancer patients than in control patients with benign breast disease (Rowinska-Zakrewska, Lazar and Burtin, 1970; Roberts, Bathgate and Stevenson, 1975).

It has also been suggested that patients with breast cancer who have increased levels of serum IgA before mastectomy have an improved prognosis (Meyer, Mackler and Beck, 1973).

Patients with breast cancer have been reported to suffer significantly less from allergic diseases than do controls without cancer (Mackay, 1966). The relationship of this observation to the pathogenesis of cancer is unknown, although there is some evidence that atopic subjects are "high responders" (*i.e.* more readily make antibodies to foreign substances than do non-atopic subjects (Platts-Mills *et al.*, 1976)).

Cancer patients were found to have slightly lower than normal IgE levels, according to single radial immunodiffusion (Augustin and Chandradasa, 1971) and so they might possibly be regarded as "low responders". However, normal IgE levels are much lower (Nye *et al.*, 1975) than was originally believed, and are below the limits of immunodiffusion procedures.

The value of previous studies has been limited by a number of factors. Firstly, no study has reported changes in serum immunoglobulin levels followed serially in the same subjects over an extended period of time. Secondly, only rarely has the extent of the disease been considered or the effects of potentially immunosuppressive therapy (*e.g.* radiotherapy or cytotoxic drugs). Finally, although the distributions of each of the serum immunoglobulin levels is log-normal, statistical comparisons have been made on data without logarithmic transformation.

This study was undertaken to determine (a) whether the serum levels of immunoglobulins IgG, IgA, IgM and IgE changed in early breast cancer compared to non-malignant breast disease: (b) whether changes in serum levels of immunoglobulins could be correlated with the spread of the tumour. The results of the first 2 years are reported here.

### PATIENTS AND METHODS

One hundred and sixty women admitted to King's College Hospital for breast tumour biopsy have been studied. Sixty-nine of these women were found to have breast cancer and 91 non-malignant breast disease.

All the women were less than 70 years of age and had breast lumps less than 5 cm in diameter, with or without palpable ipsilateral axillary glands. Those with breast cancer therefore had tumours which fell into Clinical Stage I or II of the Manchester classification (Wise, Mason and Ackerman, 1971) and had no evidence of clinically occult metastases as shown by routine chest X-ray, liver function tests and serum chemistry or bone scanning.

The 2 groups of women had venous blood sampled the day before operation, and subsequently they have had further samples taken 3 months postoperatively and then annually for between 2 and 3 years.

A careful clinical record has been kept of each cancer patient. A system for quantifying the approximate tumour mass in each cancer patient was devised to produce the clinical score, so that correlations between the immunoglobulin levels and the extent of the cancer could be performed. This scoring system was based upon the diameter of the breast tumour measured postoperatively, and additional points were given for evidence of histological spread of the tumour to involve skin, pectoral muscles or lymph nodes. Postoperatively the score was increased for local recurrence of the tumour in the scar or lymph nodes and for the development of each metastatic lesion confirmed by biopsy. Equal weighting was given to proven metastases, irrespective of site, and the score modified if a lesion increased or decreased in size. A reduced weighting was given to symptoms or investigations which suggested metastatic spread, before a lesion was confirmed (*e.g.*, persistent backache). Details of the scoring system are given in an Appendix.

Serum was separated from the blood samples, divided into aliquots, coded and stored at − 20°C. Samples were randomized and analysed in batches of 50. Estimations of immunoglobulins IgG, IgA and IgM were made, using single radial immunodiffusion on commercial Tripartigen plates (Hoechst Ltd). Total serum IgE levels were measured using radioimmunoassay as previously described in detail (Merrett and Pantin, 1975). Serum IgE levels were measured in only 100 patients preoperatively and 110 patients at follow-up.

### RESULTS

### A. *Comparison between the two diagnostic populations*

The serum levels of immunoglobulins G, A, M and E in breast cancer patients were not significantly different from the

TABLE I.—*Comparison of Serum Immunoglobulin Levels between Patients with Breast Cancer and those with Benign Breast Disease*

| | Cancer | | Benign | |
|---|---|---|---|---|
| | Mean | s.d. | Mean | s.d. |
| IgG | | | | |
| Pre-operative | 2·97 (933·4) | 0·21 | 2·96 (916·1) | 0·20 |
| 3 months | 3·07 (1174·9) | 0·18 | 3·10 (1248·8) | 0·15 |
| 1 year | 3·07 (1174·9) | 0·13 | 3·10 (1248·8) | 0·16 |
| 2 years | 2·99 (977·3) | 0·15 | 3·02 (1047·2) | 0·12 |
| IgA | | | | |
| Pre-operative | 2·32 (208·5) | 0·23 | 2·27 (184·9) | 0·25 |
| 3 months | 2·30 (198·4) | 0·26 | 2·33 (212·7) | 0·32 |
| 1 year | 2·29 (194·4) | 0·26 | 2·29 (194·4) | 0·27 |
| 2 years | 2·21 (162·2) | 0·23 | 2·21 (162·2) | 0·23 |
| IgM | | | | |
| Pre-operative | 1·95 (88·2) | 0·22 | 2·01 (101·5) | 0·23 |
| 3 months | 1·96 (90·9) | 0·29 | 2·00 (99·49) | 0·30 |
| 1 year | 2·02 (103·6) | 0·29 | 2·08 (121·5) | 0·24 |
| 2 years | 2·04 (109·7) | 0·24 | 2·10 (125·9) | 0·21 |
| IgE | | | | |
| Pre-operative | 1·39 (24·5) | 0·63 | 1·60 (40·0) | 0·68 |
| 3 months | 1·41 (25·8) | 0·58 | 1·44 (27·4) | 0·65 |
| 1 year | 1·47 (29·4) | 0·60 | 1·51 (32·5) | 0·66 |
| 2 years | 1·40 (25·1) | 0·87 | 1·32 (20·9) | 0·78 |

Mean values shown as logarithms to base 10 (antilog to base 10 of mean expressed in mg/100 ml for IgG, A and M and u/ml for IgE shown in brackets). All comparisons between Cancer and Benign are non-significant.

levels in patients with benign breast disease, either pre-operatively or at any of the follow-up times (Table I).

B. *Correlation of serum immunoglobulin levels with patients' age*

Although the mean ages of the 2 diagnostic groups differed significantly (benign group 45 years; cancer group 52 years), correlation analysis between the serum immunoglobulin level and age of the patients failed to reveal any statistically significant relationship in either group at any time.

C. *Correlation of serum immunoglobulin levels with metastatic spread*

At 24 months after mastectomy, 13 patients from the original 69 had developed definite clinical evidence of metastatic disease, and 6 patients had died of breast cancer. As expected, comparison of these patients as a group with the remaining cancer patients did not reveal statistically significant differences in their serum immunoglobulin levels at any of the follow-up times, due to their small number. However, correlation analysis between the serum immunoglobulin levels and the clinical score of each cancer patient did show a small but significant positive correlation with IgA at one year ($r = 0{\cdot}29$, $P < 0{\cdot}05$) and at 2 years ($r = 0{\cdot}26$). There was also a significant negative correlation with IgM at 3 months ($r = 0{\cdot}26$, $P < 0{\cdot}05$) (Table II).

TABLE II.—*Correlation (Pearson Coefficient, r) between Serum Immunoglobulins and Clinical Scores in Breast Cancer Patients*

| | Pre-operative correlation | Post-operative 3 month correlation | Post-operative 1 year correlation | Post-operative 2 year correlation |
|---|---|---|---|---|
| IgG | 0·05 | 0·10 | 0·25 | 0·20 |
| IgA | 0·12 | −0·20 | 0·29* | 0·26 |
| IgM | 0·09 | −0·26* | −0·05 | 0·10 |
| IgE | −0·21 | 0·02 | 0·11 | 0·13 |

* $P < 0{\cdot}05$.

FIG.—Distribution of clinical scores among the breast cancer patients at different times.

The distribution of clinical scores of the cancer patients at the various follow-up times is shown in the figure. Since so few patients have developed high scores even after 2 years, individuals with proven metastases were examined separately and the following general conclusions were drawn.

1. There was no uniform pattern of serum immunoglobulin response in patients with metastatic breast cancer.

2. Considerable rises in one or more of the serum immunoglobulin levels (from 75 to 300%), particularly IgG and IgA, but occasionally IgM and IgE, were seen in the majority of patients (9 out of 13) before the clinical appearance of their metastases.

3. Preoperative elevation of serum IgG level ($> 2$ g/100 ml) was usually associated with early metastatic disease (4/5 patients).

4. Preoperative elevation of serum IgA level ($> 450$ mg/100 ml) was rarely associated with metastatic disease in the first 2 years (1/7) particularly if the serum level fell progressively.

D. *The effects of therapy on serum immunoglobulin levels*

The patients with breast cancer were allocated at random to one of 2 treatment groups in the King's/Cambridge breast trial (Baum, Edwards and Magarey, 1972), and 25 patients received routine postoperative radiotherapy to the ipsilateral axillary nodes between the time of mastectomy and the 3-month follow-up sample. These patients as a group had significantly lower mean serum IgM and IgE levels preoperatively, and also significantly lower mean serum IgM levels at 3 and 12 months postoperatively, than in breast cancer patients who did not receive radiotherapy (Table III). There was no evidence that the group not receiving radiotherapy were clinically more advanced than those who did not. (No IgE levels were measured at 2 years postoperatively.)

Individual patients who subsequently developed metastatic breast cancer received various additional forms of therapy, including radiotherapy, chemotherapy and hormone-replacement therapy. Although reductions in some of the immunoglobulin levels were seen, especially in serum IgG, it was not possible to separate the effects of the therapy from those of the disease process.

### DISCUSSION

This study has not demonstrated any alteration in the levels of serum immunoglobulins IgG, IgA, IgM or IgE in patients with breast cancer relative to patients with benign breast lumps, when followed prospectively for 2 years. We have not,

TABLE III.—*Effect of Radiotherapy on Serum Immunoglobulin Levels in Breast Cancer Patients*

| Radiotherapy group | Serum immunoglobulin | Number of cases | Mean | s.d. | P |
|---|---|---|---|---|---|
| | Pre-operative | | | | |
| Without | IgG | 42 | 3·01 (1012·4) | 0·21 | NS |
| With | | 25 | 2·93 (845·5) | 0·21 | |
| Without | IgA | 42 | 2·33 (214·8) | 0·24 | NS |
| With | | 25 | 2·29 (194·4) | 0·22 | |
| Without | IgM | 42 | 1·98 (96·5) | 0·24 | 0·027 |
| With | | 25 | 1·88 (75·2) | 0·16 | |
| Without | IgE | 28 | 1·56 (36·3) | 0·55 | 0·004 |
| With | | 11 | 0·94 (8·8) | 0·60 | |
| | At 3 months | | | | |
| Without | IgG | 40 | 3·05 (1130·1) | 0·15 | NS |
| With | | 20 | 3·10 (1248·8) | 0·22 | |
| Without | IgA | 40 | 2·28 (188·7) | 0·27 | NS |
| With | | 20 | 2·33 (212·7) | 0·25 | |
| Without | IgM | 40 | 2·01 (101·5) | 0·31 | 0·054 |
| With | | 20 | 1·85 (71·5) | 0·22 | |
| Without | IgE | 32 | 1·48 (30·3) | 0·51 | NS |
| With | | 12 | 1·21 (16·1) | 0·71 | |
| | At 1 year | | | | |
| Without | IgG | 28 | 3·08 (1211·8) | 0·13 | NS |
| With | | 19 | 3·08 (1199·9) | 0·12 | |
| Without | IgA | 28 | 2·29 (194·4) | 0·28 | NS |
| With | | 19 | 2·28 (192·4) | 0·25 | |
| Without | IgM | 28 | 2·08 (121·5) | 0·29 | 0·031 |
| With | | 18 | 1·90 (79·8) | 0·23 | |
| Without | IgE | 28 | 1·55 (35·9) | 0·60 | NS |
| With | | 15 | 1·30 (19·9) | 0·58 | |
| | At 2 years | | | | |
| Without | IgG | 26 | 2·99 (982·4) | 0·14 | NS |
| With | | 16 | 2·98 (963·0) | 0·16 | |
| Without | IgA | 26 | 2·16 (145·5) | 0·21 | NS |
| With | | 16 | 2·28 (188·7) | 0·24 | |
| Without | IgM | 26 | 2·05 (111·1) | 0·25 | NS |
| With | | 16 | 2·02 (103·6) | 0·21 | |

Mean values shown as logarithms to base 10 (antilog of mean to base 10 expressed in mg/100 ml for IgG, A and M and u/ml for IgE shown in parentheses).
NS = Not significant.

therefore, confirmed the finding of elevated serum IgA levels in breast cancer patients reported by Rowinska-Zakrewska *et al.* (1970) and by Roberts *et al.* (1975). However, the patients in our study were especially selected to be at the earliest clinical stage of their disease and, since serum IgA levels may rise with advancing disease, this difference in the selection of patients, plus the statistical analysis on logarithmically transformed data, may account for our differing results.

The model IgE value in a non-atopic population was found to be 21 u/ml (Nye *et al.*, 1975) and 38 u/ml in a population that excluded asthma and chronic bronchitis sufferers (Burr *et al.*, 1975).

The modal value obtained in this study are not significantly different, although the number of breast cancer patients with a history of atopic allergies (6/69) was significantly lower than the control group (20/91) ($P < 0{\cdot}05$). There is therefore no diagnostic value in measuring the levels of serum immunoglobulins in patients with breast tumours.

However, the measurement of serum immunoglobulin levels in breast cancer patients may be of some value as an indicator of tumour spread. There is a positive correlation between serum IgA level and the advancement of metastatic breast cancer, as quantified by the clinical score. Analysis of individuals who have subsequently developed metastases confirms that large rises in serum immunoglobulins, particularly IgG and IgA, may antedate the detection of metastases. At present it is impossible to detect, let alone quantify, subclinical spread of breast cancer, and the present scoring must clearly underestimate the tumour mass of many patients, particularly in the first few postoperative months. Subsequently, the effects of the advancing disease and coincident therapies upon the serum immunoglobulin levels may be impossible to differentiate.

The association of advancing metastatic breast cancer with rises in serum immunoglobulin levels of all major classes, but particularly IgA and IgG, suggests a defence reaction against increasing tumour load or the secretion of immunoglobulins by the tumour.

The first hypothesis is supported by our previous finding of a highly significant correlation between preoperative serum IgA and IgM levels and the degree of mononuclear infiltrate of the primary breast tumour ($r = 0{\cdot}40$ and $0{\cdot}30$ respectively) (Tee and Pettingale, unpublished).

In addition, we have found diverse changes in other serum proteins in our patients with breast cancer (Pettingale and Tee, 1977) which are more likely to result from an indirect effect of the tumour.

The authors would like to thank Patricia White and Kevin Ryan for their help with the statistical analysis; they are grateful to the patients for their willing cooperation and to their colleagues on the Faith Courtauld Unit for Human Studies in Cancer at King's College Hospital Medical School for their help.

### APPENDIX

### *Clinical Scoring System*

*General Instructions*

(1) The score is a system for quantifying the approximate total tumour mass in a patient with breast cancer at different times.

(2) The score should be calculated prospectively and corrected retrospectively when confirmatory investigations have established or refuted progression of the disease. This particularly applies to Section II (ii, iii and iv). Only when metastatic spread has been confirmed and there is measurable evidence that spread had occurred before this confirmation should the additional scores under these sections be allocated.

(3) If any clinical, biochemical or other feature could be explained by any non-tumour pathology, no score should be allocated.

(4) Change in size of existing lesions should only be based on objective measurements.

| I. *Pre-operative* | Score |
|---|---|
| (a) *Local* | |
| Initial tumour diameter (operative specimen) in cm | +1 per cm |
| Histological evidence of superficial or deep involvement (either/both) | +1 |
| Histological node involvement | +1 for each anatomical region |
| (b) Add score of any *systemic* involvement (as below) | |
| (c) After operation subtract, the *local* score | |

II. *Post-operative*

(a) *Local.* Persistence/recurrence in node, scar etc. +3

(b) *Systemic*

(i) *Confirmed* (*e.g.* biopsy/X-ray/cytology)

| | |
|---|---|
| Bone | +5 |
| Lung | +5 |
| Liver | +5 |
| Other (*e.g.* marrow) | +5 |

(ii) Suspected symptoms/clinical examination non-specific investigations (*e.g.* radioisotope scanning)

| | |
|---|---|
| Bone | +3 |
| Lung | +3 |
| Liver | +3 |
| Other | +3 |

(iii) Unexplained biochemical abnormality alone (elevated enzymes) +1

(iv) Additional general symptoms (*e.g.* weight loss, fever, malaise, anaemia, etc.) +1

III. *At each continuing assessment*

| | |
|---|---|
| (a) New system confirmed | +5 |
| (b) New system suspected | +3 |
| (c) Increase in size of existing lesion (for each) | +1 |
| (d) Decrease in size of existing lesion | −1 |
| (e) No change in size | 0 |

*Example*

*Pre-operative.*—Woman with 5 cm tumour with superficial involvement + tumour in nodes. Raised serum LDH.
*Score:* 5 + 1 + 1 + 1 = 8

*Post-operative score.*—+ 1 *to carry forward*

| | | | |
|---|---|---|---|
| At 3-months follow-up: | LDH higher, but well | +1 | *Total* +2 |
| At 6-months follow-up: | backache, X-ray, —ve | +3 | *Total* +5 |
| At 9-months follow-up: | X-rays now show lytic lesions, | +2 | (to make confirmed score) |
| | alkaline phosphatase now raised, and LDH raised more | +1 | *Total* +8 |
| At 10-months follow-up: | radiotherapy | | |
| At 12-months follow-up: | backache better | | |
| | lesions smaller | −1 | |
| | serum enzyme levels down | −1 | *Total* +6 |
| At 15-months follow-up: | dyspnoea | | |
| | CXR shows pleural effusion oophorectomy | +5 | *Total* +11 |
| At 16-months follow-up: | no change | 0 | *Total* +11 |
| At 18-months follow-up: | cytotoxics into pleural effusion responds | −1 | *Total* +10 |
| At 20-months follow-up: | very ill—fever, weight loss, etc. | +1 | *Total* +11 |
| At 21 months: | dies. | | |

### REFERENCES

AUGUSTIN, R. & CHANDRADASA, K. S. (1971) IgE Levels and Allergic Skin Reactions in Cancer and Non-cancer Patients. *Int. Arch. Allergy appl. Immunol.*, **41**, 141.

BAUM, M., EDWARDS, M. H. & MAGAREY, C. J. (1972) Organisation of Clinical Trial on a National Scale: Management of Early Cancer of the Breast. *Br. med. J.*, iv, 476.

BURR, M. L., ST LEGER, A. S., BEVAN, C. & MERRETT, T. G. (1975) A Community Survey of Asthmatic Characteristics. *Thorax*, **30**, 663.

HUGHES, N. R. (1971) Serum Concentrations of $\gamma_G$, $\gamma_A$ and $\gamma_M$ Immunoglobulins in Patients with Carcinoma, Melanoma and Sarcoma. *J. natn. Cancer Inst.*, **46**, 1015.

MACKAY, W. D. (1966) The Incidence of Allergic Disorders and Cancer. *Br. J. Cancer*. **20**, 434.

MERRETT, T. G. & PANTIN, C. F. A. (1975) Increasing the Precision and Speed of the Separation Step in Radioimmunoassays. *Clin. chim. Acta.*, **65**, 131.

MEYER, K. K., MACKLER, G. L. & BECK, W. C. (1973) Increased IgA in Women Free of Recurrence after Mastectomy and Radiation. *Arch. Surg.*, **107**, 159.

NYE, L., MERRETT, T. G., LANDON, J. & WHITE, R. (1975) A Detailed Investigation of Circulating IgE Levels in a Normal Population. *Clin. Allerg.*, **5**, 13.

PETTINGALE, K. W. & TEE, D. E. H. (1977) Serum Protein Changes in Breast Cancer: a Prospective Study *J. clin. Path.*

PLATTS-MILLS, T. A. E., VON MAUR, R. K., ISHIZAKA, K., NORMAN, P. S. & LICHTENSTEIN, L. M. (1976) IgA and IgG Anti-ragweed Antibodies in Nasal Secretions: Quantitative Measurements of Antibodies and Correlation with Inhibition of Histamine Release. *J. clin. Invest.*, **57**, 1041.

ROBERTS, M. M., BATHGATE, E. M. & STEVENSON, A. (1975) Serum Immunoglobulin Levels in Patients with Breast Cancer. *Cancer, N.Y.*, **36**, 221.

ROWINSKA-ZAKREWSKA, E., LAZAR, P. & BURTIN, P. (1970) Dosage des Immunoglobulines dans le Sérum des Cancéreux. *Ann. Inst. Pasteur*, **119**, 621.

WISE, L., MASON, A. Y. & ACKERMAN, L. V. (1971) Local Excision and Irradiation: an Alternative Method for the Treatment of Early Mammary Cancer. *Ann. Surg.*, **174**, 392.